# Supplementary material for: Adolescent abortion care trajectories and safety in Ethiopia, Malawi, and Zambia: A comparative mixed methods study
Source: PLOS Glob Public Health. 2025 May 6;5(5):e0004469. doi: 10.1371/journal.pgph.0004469 (PMC12054873; doi:10.1371/journal.pgph.0004469)
Supplement: Attachment — Submitted filename: Response to reviewers_v3.docx [file pgph.0004469.s002.docx]

| **Comment** | **Response (and any manuscript change)** |  |
| --- | --- | --- |
| Reviewer 1 | | |
| Abstract has minor grammar and spelling mistakes | Our apologies. The abstract has been reviewed and corrected. |  |
| Well written manuscript | Thank you. |  |
| Verbatim on pills and post abortion contraception is not child friendly. It involves questioning a child on why she has not asked about the injection that was given to her. Please review | We understand the importance of child-friendly language. Our research assistants were trained in adolescent-friendly communication, ensuring they used simple, clear, non-judgmental, and supportive language. Reflecting our focus on adolescents’ experiences of abortion-related care, our questions about post-abortion contraception focused on adolescents’ experiences. For example, whether she had been given an opportunity to ask questions about the contraceptive that was provided. Reflecting our methodology of a qualitative interview with a semi-structured question guide, adolescents were not interrogated about why they had not asked questions of their provider. Rather, if an adolescent mentioned that she had been offered – and received – contraception, she was asked if she had had an opportunity to ask questions, or whether she had been offered any alternate contraceptive methods. Our approach to the design and implementation of our research is grounded in principles of evolving adolescent capacity, including to consent to sex and abortion-related care, and to assent or consent to participate in research. |  |
| Is health professions education in the study areas "gendered"? Please comment in discussion | Our study did not set out to assess the education of health professionals. Our data were collected from adolescents after their care had been provided, sometimes by one and often by teams of healthcare workers, and we did not attempt to determine the roles, professions or genders of the health workers. We have added a sentence to the Discussion: *Our analyses suggest that education and training for healthcare workers involved in the care of adolescents needs to be adolescent-centred and non-stigmatising, reflecting findings elsewhere.* |  |
| Reviewer 2 | | |
| I commend the authors for this study, which addresses critical issues in adolescent reproductive health, specifically in the context of legally diverse African countries. Its focus on abortion trajectories across Ethiopia, Malawi, and Zambia offers important insights into the effects of legal, social, and healthcare contexts on the safety of adolescent abortion experiences. The use of a comparative mixed-methods approach adds depth to the findings, which highlight the implications of restrictive versus permissive abortion laws and the resulting health and social outcomes for adolescents, which has substantial policy relevance. This study makes a valuable contribution to understanding adolescent abortion care in varied legal contexts. With minor revisions to clarity, methodology, and ethics, this paper is suitable for publication in PLOS Global Public Health | Thank you |  |
| Abstract and Key Findings: The abstract could benefit from concise language and clearer presentation of key findings, particularly in terms of statistical results. Additionally, a summary of policy implications would strengthen the abstract. | The abstract has been revised to include numbers (and %).  New sentence added to highlight the policy implications: *Our analyses show that less restrictive abortion policies and accessible abortion services improve adolescent chances to access abortion care.* |  |
| Detailed Methodology Explanation: While the methods are robust, more details on sampling and potential biases, especially regarding urban-only recruitment, would improve transparency. | New text added to methodology to clarify our purposive approach to sampling: *Our approach to sampling was purposive and facility-based, with recruitment from urban public clinics and wards that provided abortion-related care at each study facility.*  Our limitations discussion acknowledges our exclusion of rural study sites, and therefore the experiences of rural adolescents. Our recruitment sites were all urban, however, some rural adolescents travelled substantial distances to seek care at these urban facilities, some of which were referral facilities. |  |
| Data Analysis: The paper could be strengthened by including effect sizes and confidence intervals for quantitative results where relevant. This addition would help readers better gauge the practical significance of findings. | Country-level statistically significant associations have been added to the text; non-significant associations are noted. Effect sizes and standard errors have been added to tables, as appropriate. Interquartile ranges have been added to medians, as appropriate. |  |
| Discussion of Limitations: Although limitations are mentioned, expanding on how urban-centric sampling and potential recall bias may affect the generalizability of findings would enhance the study’s transparency. | New text added:  *Our purposive sample selection may lead to selection bias. Given the urban study sites, rural participants were underrepresented in our study, which may limit the statistical generalisability of our findings. Some rural adolescents travelled substantial distances to access care in our urban study facilities. Some rural adolescents travelled substantial distances to access care in our urban study facilities. However, adolescents in our sample were socio-economically diverse, and abortion care is geographically centralised, especially in Malawi and Zambia.*  Recall and social desirability biases are already included in our limitations section. The timing of our interviews, soon after adolescents had completed their treatment and were ready for discharge, sought to mitigate recall bias. Our research assistant training included in-depth attention to adolescent-friendly and non-judgmental approaches to interviewing to reduce social desirability bias. Our urban recruitment facilities might contribute to a cluster effect due to our facility-based recruitment. However, in Malawi and Zambia choices for public facility abortion care were very limited for both urban and rural adolescents, likely resulting in a wide range of socio-demographic characteristics and travel distances as many adolescents mentioned seeking care at these specific sites due to confidentiality concerns in their communities, if care was available there. |  |
| Ethical Considerations: Greater detail on how sensitive information was safeguarded, given the vulnerability of the adolescent population, would strengthen the ethical considerations of the study. | New text added: “*Data security measures included: hard copy forms kept in a locked cabinet in a locked office; deletion of audio recordings once uploaded to an encrypted and password-protected laptop; post-study deletion of all files from study laptops; audio recordings deleted after quality checks completed; and, use of a secure service for transfer of files to an encrypted server.”*  And  *“Interviews were conducted in private offices.”*  Additional information for the reviewer – journal word limits preclude us adding this level of details: Research assistants were trained in data security and privacy. The participant information sheet made no reference to abortion. All of the open access data have been carefully checked to remove all direct and indirect identifiers. |  |
| Reviewer 3 | | |
| Responded “No” to Q3 on data availability | Our data – quantitative and qualitative – are publicly available here: <https://doi.org/10.5255/UKDA-SN-856965> |  |
| I suggest that the authors expand their analysis to include an investigation of the complications associated with abortion and an evaluation of the mental stress experienced by adolescents. These areas of focus would be more appropriate and would significantly enhance the quality of the article. | Thank you for your thoughtful suggestion. We agree that these are important areas of focus for future research. Our cross-sectional study did not set out to assess the mental health sequelae of abortion-related care-seeking or the severity of complications for which post-abortion care was sought. As such, we disagree that these areas of focus are “more appropriate” than the focus topics in our article, which seeks to understand adolescent trajectories to, and experiences of, abortion-related care in the three countries. Inclusion of an in-depth analysis of mental stress and complications would require a significant shift in the scope and methodology of the study. However, we acknowledge the importance of these factors and suggest them as valuable areas for future research. |  |
| Would you clarify the sample formula and how did you come up with 313 cases? | Thank you for your comment and question regarding the sample size. We would like to clarify that, as a mixed methods observational descriptive study – generating qualitative and quantitative evidence – we did not use a formal sample size formula. Instead, our sample size reflects two key factors 1) The need to balance sufficient observations across three countries for descriptive quantitative analyses with the requirements of qualitative data to identify when saturation has occurred. 2) The capacity (financial, human etc.) to conduct the study. |  |
| Would you specify how did you chose which healthcare facilities to recruit the adolescent? and your specific effort to reduce the possible selection bias? | Facilities were selected on the basis of three criteria: located in a major urban city; known to be a public sector provider of abortion-related care (safe abortion or post-abortion care) in that context; team member professional association with the facility. In two (Malawi, Zambia) of the three countries the choices for care in the public sector were very limited. This type of care, while often straightforward, is not routinely or systematically available in all public sector facilities. In addition, the study facilities needed to have sufficient caseloads so that we could collect data over a limited number of months due to resource constraints.  To reduce potential selection bias was reduced through the application of our inclusion criteria: adolescents aged 10-19 years seeking abortion-related care (either safe abortion or care for an abortion initiated elsewhere). These inclusion criteria reflected our research aim of understanding diverse adolescent trajectories to abortion-related care in the three countries. The study facilities were located in areas serving diverse groups. |  |
| What is the ratio of public vs. private healthcare facilities allowed to provide abortion services in those countries? | Data do not exist for each of the three study countries that would allow for an answer to this specific question. Table 1 describes – with sources - the public/private provision of abortion services in each country. As shown in our analyses, many adolescents self-manage their abortion using medication abortion. A focus only on formal facilities – public and private – ignores this critical dimension of adolescent abortion-related care-seeking. Additionally, the definition of “private” care is challenging; private abortion providers in the three countries might be unauthorised, not standardized in terms of care or service, untrained and not monitored for quality of care or adverse outcomes. |  |
| What are your inclusion and exclusion criteria? | Inclusion: Adolescents aged 10-19 years who sought abortion-related care (either safe abortion or post-abortion care for an abortion initiated elsewhere) and consented (>18y) or assented (<18) to participate in the study.  Exclusion criteria: People aged below 10 years or above 19 years; not seeking abortion-related care; did not consent or assent to participate in the study. |  |
| Would you specify the definition of safety of abortion? It seems confusing for me and relies on subjects' experiences which might be very biased. | Our analyses developed categories of safety, incorporating adolescents’ subjective experiences, to reflect the continuum of abortion safety [rather than a binary of un/safe] and incorporate the use of self-managed medication abortion by some adolescents. In our analyses, ‘abortion safety’ (safe versus less or least safe) was determined using biomedical classifications and classified for each participant based on their interview self-reports and [medical records](https://www.sciencedirect.com/topics/medicine-and-dentistry/medical-record) (where available at time of interview). ‘Safe’ abortions were classified as those for which adolescents received medically recommended care (medication or procedural abortion) within a clinical setting. Self-use of medication abortion was categorized as ‘less safe’ and was based on adolescent’s descriptions of their experiences and the drugs they used outside clinical settings. “Least safe” abortions included the use of all other (non-medication abortion) pharmaceuticals and/or other toxins, objects inserted in the uterus, or herbal [abortifacients](https://www.sciencedirect.com/topics/medicine-and-dentistry/abortifacient). |  |
| Statistical analysis section were missing. | Reflecting our methodology, we present bivariate descriptive data from our observational study, highlighting the relationships between key variables. |  |
| Why did you quote the subjects responses? It is helpful not informative. | We present mixed methods – quantitative and qualitative – data. It is standard practice in the field, and for the journal PLoSGPH, to include illustrative quotes from qualitative data to evidence analyses. The absence of adolescent voices and their abortion experiences in the literature is a key gap that our research helps to fill. |  |
| The results section needs to be rewrote with more appropriate, systematic, and statistical way. | As the study involves quantitative and qualitative data, we have presented the quantitative data in a descriptive format using cross-tabulations to clearly show the relationships between key variables. Additionally, we have added country-level statistically significant associations to the text to emphasise the meaningful differences across the three study countries. To enhance the statistical rigour, we have included effect sizes and standard errors in the tables where appropriate. Furthermore, the structure of our results section is systematic, aligning with the flow of our data and analyses. We have organized the results to reflect the different stages of adolescents’ abortion-related care trajectories and experiences, from delays in care-seeking to post-abortion contraception, ensuring that the presentation follows a logical sequence. |  |
| Discussion: This section is overwhelming due to unnecessary discussion that does not focus on the results presented in the article. I suggest that the author restructure it into 3-4 paragraphs, each addressing a specific topic: the safety and complications of abortion, delays in seeking abortion, and potential future policy recommendations. | We appreciate your suggestion to restructure the discussion into 3-4 paragraphs. However, we believe that the current structure effectively serves the purpose of the discussion by addressing the four key components: 1) Interpretation of results 2) Comparison with previous studies 3) Implications 4) Limitations. Given the broad scope of our study, spanning three diverse countries, we feel the discussion needs to address various interconnected aspects, including the abortion safety and complications, delays in seeking abortion, and potential policy recommendations. Rather than breaking these into separate sections, we have integrated them in a way that reflects the complexity of the topic and presents a comprehensive interpretation of our findings. Our discussion is concise and systematic and values the time of adolescents that participated in the study. We are confident that this structure allows for a more nuanced interpretation, highlighting the implications of our findings for adolescents, research, and policy in a cohesive manner. |  |
| Line 36. Typo. "....and Zambia between 2018-19l" | Corrected. |  |
| Lines 34-37. The methods was not well described. Would you elaborate more details about your study design including time (month/year), location (which locations have you collected the data?), inclusion and exclusion criteria... | Abstract amended to include location (city) and month/year. Per request we have clarified in/exclusion criteria for adolescents and provided more detail about facilities selection. |  |
| Lines 41-49. The result might need to include the number (percentage). | Numbers added |  |
| The authors might need to restructure the manuscript as Introduction - Methods - Results - Discussion -Conclusion and use subheading as needed. | Thank you. “CONTEXT” changed to “Context” and made a subhead in the Introduction. Our manuscript already uses the structure of Introduction - Methodology - Results - Discussion – Conclusion. |  |
